# Bidirectional Associations of Depressive Symptoms and Cognitive Function Over Time

**DOI:** 10.1001/jamanetworkopen.2024.16305

**Published:** 2024-06-11

**Authors:** Jiamin Yin, Amber John, Dorina Cadar

**Affiliations:** 1 Department of Epidemiology and Public Health, University College London, London, United Kingdom; 2 Department of Behavioural Science in Health, University College London, London, United Kingdom; 3 Department of Public Health Sciences, University of Rochester Medical Center, Rochester, New York; 4 ADAPT Lab, Research Department of Clinical, Educational and Health Psychology, UCL, London, United Kingdom; 5 CEDAR Lab, Department of Neuroscience, Brighton and Sussex Medical School, Sussex, United Kingdom

## Abstract

**Importance:**

Cognitive decline and depressive symptoms often co-occur among older adults, and they share several mechanisms. Despite the fact that cognitive dysfunction has been linked to increased depressive symptoms, the directionality of this association remains unclear.

**Objective:**

To examine whether there is a bidirectional association between depressive symptoms and cognitive function in English adults aged 50 years or older throughout a 16-year follow-up period.

**Design, Setting, and Participants:**

This cohort study included a nationally representative sample of community-dwelling English adults aged 50 years or older. The current analysis included 8268 eligible participants with relevant data. These participants were examined every other year from 2002 and 2003 until 2018 and 2019, resulting in a follow-up period of up to 16 years. Data were analyzed from July to November 2023.

**Main Outcomes and Measures:**

The bivariate dual change score models were used to estimate the multivariable associations between depressive symptoms and cognitive function, which were interchangeably used as exposures and outcomes. Cognitive measures include memory and verbal fluency tests, while the Center for Epidemiologic Studies Depression Scale evaluated depressive symptoms.

**Results:**

The study population of 8268 participants had a mean (SD) age of 64 (10) years at the study baseline, and 4517 participants (55%) were female. Higher depressive symptoms were cross-sectionally associated with poorer memory (β intercept, −0.018; standard error [SE], 0.004; *P* < .001) and verbal fluency (β intercept, −0.009; SE, 0.004; *P* = .02) at study baseline. A steeper linear change in depressive symptoms was associated with an accelerated memory change (β intercept, −0.253; SE, 0.079; *P* = .001), and a linear change in memory was associated with an acceleration in depressive symptoms over time (β intercept, 0.016; SE, 0.006; *P* = .005). This bidirectional change was not observed with verbal fluency.

**Conclusions and Relevance:**

In this study, greater depressive symptoms were associated with poorer memory at the study baseline and steeper memory change over time. A gradual linear change in depressive symptoms contributed to accelerated memory loss and vice versa, suggesting that psychological mood and memory performance are intrinsically associated.

## Key Points

**Question:**

Are depressive symptoms associated with cognitive function among adults aged 50 years or older?

**Findings:**

In this longitudinal analysis of 8268 eligible participants, greater levels of depressive symptoms at study baseline and an accelerated change in depressive symptoms over time were associated with faster memory decline. In reverse, a steeper change in memory was also reciprocally associated with a more rapid change in depressive symptoms over time.

**Meaning:**

These findings suggest that changes in depressive symptoms are associated with cognitive performance.

## Teaser

This cohort study examines depressive symptoms and cognitive function in English adults aged 50 years or older throughout a 16-year follow-up period and evaluates whether there is a bidirectional association.

## Introduction

Subtle cognitive decline can be observed as a result of age in most older adults.^1^ However, if cognitive deficits are present in 1 or more domains and negatively impact daily life, it could lead to mild cognitive impairment (MCI) or dementia.^2,3^ Due to comorbidity or severe life events like bereavement, depressive symptoms are also common among older adults. As a result of high underdiagnosis, a significant percentage of patients with depression are left untreated and living with a variety of symptoms.^4^ Cognitive decline and depressive symptoms share some common features and regularly co-occur among older adults.^5^ Depression in early life was shown to be a risk factor for dementia, and depression in later life can be considered a prodrome of dementia.^6,7,8^ In contrast, cognitive dysfunction or dementia could also be attributable to depressive symptoms.^9^ This indicates that these are not mutually exclusive and that there may be a bidirectional association.

To our knowledge, 3 epidemiologic studies have attempted to investigate the bidirectional association between depressive symptoms and cognitive function among older adults, but the findings are mixed. One study^10^ identified a bidirectional association between depressive symptoms and MCI throughout a 20-year follow-up, while another study^11^ conducted in the UK reported no significant association in any direction during a 6-year follow-up. However, both failed to account for the dual changes in depressive symptoms and cognitive function by analyzing different directions separately. The third study found a mutual association between depressive symptoms and cognitive decline, using latent growth curve modeling.^12^ By using a global score to measure cognition, these studies may have overlooked essential variations in the rate of change and nuances in the nature of the association with depressive symptoms across cognitive domains. Furthermore, since a bidirectional association between loneliness and memory function has been identified,^13^ these results may be confounded by loneliness. The present study aimed to examine the bidirectional association of depressive symptoms and cognitive function during a 16-year follow-up by conducting parallel bivariate analyses in a nationally representative sample of the English population aged 50 years and older living in the community.

## Method

### Study Population

Data were drawn from the English Longitudinal Study of Aging (ELSA), a panel study of a nationally representative sample of adults aged 50 years and older. ELSA collects data on of topics, such as health, socioeconomic status, psychological conditions, and cognitive function, every other year. A detailed study description can be found elsewhere.^14^ In this analysis, the study baseline was considered the first wave (2002 to 2003), and participants were followed up to wave 9 (2018 to 2019), which was the latest wave of data available at the time of these analyses, leading to 16 years of follow-up. Data at later waves were collected during the COVID-19 pandemic; hence, they were excluded to avoid the potential confounding impact of the pandemic.

**Figure 1.**
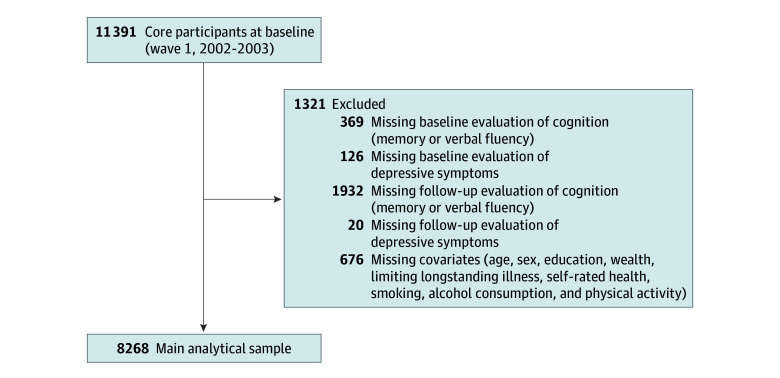
Flowchart of the Selection Process of the Analytical Sample in ELSA ELSA indicates English Longitudinal Study of Aging.

The study population was confined to the 11 391 core members at the study baseline. We excluded those who had no data at the study baseline or at least 1 follow-up evaluation of cognitive function or depressive symptoms. There were 8268 individuals remaining in the analysis. Figure 1 presents our analytic sample.

The National Research Ethics Service granted ethics approval for each ELSA wave. All participants provided informed consent. This cohort study followed Strengthening the Reporting of Observational Studies in Epidemiology (STROBE) reporting guideline.

### Main Outcomes and Measures Ascertainment

#### Cognitive Functioning

Memory was assessed via memory recall tests of 10 unrelated and randomly assigned words.^15,16^ A combination of the numbers of words recalled immediately and after a short delay was used as a continuous measure of memory, leading to a possible score ranging from 0 to 20. Verbal fluency was assessed using the animal naming test.^17^ The total number of nonrepeated animal names produced represented a continuous score of verbal fluency. Data for verbal fluency at wave 6 were not available. This analysis used continuous scores of both cognitive tests, with higher scores indicating a higher level of mental functioning.

#### Depressive Symptoms

Self-reported depressive symptoms during the week prior to interviews were assessed since wave 1 using the 8-item version of the Centre for Epidemiologic Studies Depression Scale (CES-D).^18^ Each question was coded as 0 (ie, no) or 1 (ie, yes), resulting in a summary score ranging from 0 to 8, with higher scores indicating greater severity of depressive symptoms. The 8-item CES-D has been validated against the 20-item version and is widely used in aging research. It also shows good consistency across waves (α = 0.68).^19^ The summary score was log-transformed before being added to models due to its highly skewed distribution. Depressive symptoms were examined both as an estimator and an outcome of memory and verbal fluency during a 16-year follow-up.

#### Covariates

Demographic factors (age, sex, education, and wealth) were included in the study baseline (wave 1). Educational attainment was categorized into 3 levels: low (compulsory schooling), medium (up to high school diploma), and high (university degree or higher), based on the highest qualifications obtained. Total wealth includes the value of the respondent’s home, financial assets, and physical wealth, which was divided into tertiles. Information about health status was also collected by asking participants if they had any illness or disability that impaired their everyday life over an extended period. Self-rated overall health was dichotomized (fair or worse health vs good or better health). Lifestyle factors included smoking (current smoker vs nonsmoker), alcohol consumption (daily vs less than daily) and physical activity (mild or below vs moderate or above).

### Statistical Analysis

All variables were descriptively analyzed by wave. We also compared the study baseline characteristics of participants with and without complete data during the study period. A set of bivariate dual change score models was conducted to assess the cross-sectional association between depressive symptoms and cognitive function at the study baseline and the dual parallel changes between depressive symptoms and cognitive function during follow-up. Maximum likelihood robust estimation was applied during the modeling process, which generates unbiased estimates under the assumption of missing at random. Memory and verbal fluency were modeled separately.

The variable for time in the analysis was calculated as years since the study baseline, presenting the changes in cognitive function and depressive symptoms every 2 years during the follow-up. The follow-up period was up to 16 years when exploring the association of depressive symptoms with memory and up to 8 years for the investigation of verbal fluency. All statistical models adjusted for age, sex, education, wealth, limiting long-standing illness, self-rated health, smoking, alcohol consumption, and physical activity. For the purpose of interpretation, the age was centered at 65 years. Bayesian information criteria were used for model selection, and based on this, we selected to report the current results indicating a quadratic function of change. The outputs of these models represent the following:

1. The estimated average level at the study baseline, the rate of change over time in both linear and quadratic terms for depressive symptoms and cognitive scores;
2. The association between the study baseline level and the speed of change in each outcome;
3. The impact of covariates on the intercept and linear change in each outcome;
4. The cross-sectional associations between depressive symptoms and both domains of cognitive function at the study baseline;
5. The prospective impact of depressive symptoms at the study baseline on changes in each domain of cognitive function and the prospective impact of each measure of cognitive function at the study baseline on changes in depressive symptoms;
6. Bivariate dual change parallel associations between the linear slope in depressive symptoms and nonlinear (quadratic) changes in each cognitive domain and between the linear slope in each cognitive domain and the quadratic slope of depressive symptoms.

We performed 3 sets of sensitivity analyses to assess the robustness of our results. We first removed the question about loneliness to exclude the potential overlapping impact of loneliness on cognitive functioning. Another study was conducted to examine whether those with low cognition drive the observed association by excluding those whose cognition measure at the study baseline was in the lowest quintile. We finally repeated the analysis after censoring individuals with diagnoses of stroke or dementia. Dementia occurrence was determined at each wave, using an algorithm based on a combination of self-reported or informant-reported physician diagnosis of dementia or Alzheimer disease or an informant score above the threshold of 3.38 on the 16-question Informant Questionnaire on Cognitive Decline in the Elderly.^20,21,22,23^ The hypotheses tests were 2-sided, and a significance level of *P* <  .05 was used. All data analyses were conducted using MPlus version 7 (MPlus Software). Data were collected from 2002 to 2019 and analyzed from July to November 2023.

## Results

The study sample included 8268 eligible participants. Table 1 presents the characteristics of the sample during the study period. At wave 1, the sample had a mean age of 64 (10) years. Among all participants, 3751 (45%) were males, 4517 (55%) were females, and 2070 (25%) had high educational levels.

**Table 1.** Psychosocial and Demographic Characteristics of the Sample at Each Wave of the ELSA

Variables	Participants, No. (%)								
	Wave 1 (2002-2003)	Wave 2 (2004-2005)	Wave 3 (2006-2007)	Wave 4 (2008-2009)	Wave 5 (2010-2011)	Wave 6 (2012-2013)	Wave 7 (2014-2015)	Wave 8 (2016-2017)	Wave 9 (2018-2019)
Participants, No.	8268	7841	6801	5988	5653	5137	4457	3857	3343
Memory, mean (SD) [range]	9.8 (3.4) [0-20]	10 (3.6) [0-20]	10 (3.7) [0-20]	10 (3.7) [0-20]	10 (3.8) [0-20]	10 (3.8) [0-20]	9.9 (3.8) [0-20]	9.5 (4.4) [0-20]	9.9 (3.8) [0-20]
Verbal fluency, mean (SD) [range]	20 (6.2) [0-50]	20 (6.5) [0-63]	20 (6.7) [0-56]	20 (6.9) [0-54]	20 (6.9) [0-51]	NA [NA]	20 (7.3) [0-67]	20 (7.4) [0-61]	21 (7.6) [0-49]
Depressive symptoms, median (IQR) [range]	1 (0-2) [0-8]	1 (0-2) [0-8]	1 (0-2) [0-8]	1 (0-2) [0-8]	1 (0-2) [0-8]	1 (0-2) [0-8]	1 (0-2) [0-8]	1 (0-2) [0-8]	1 (0-2) [0-8]
Age, y									
Mean (SD)	64 (9.8)	67 (9.9)	68 (9.7)	70 (9.4)	70 (8.1)	72 (7.6)	74 (8.4)	75 (7.9)	76 (7.1)
Sex									
Male	3751 (45)	3553 (45)	3051 (45)	2667 (45)	2505 (44)	2273 (44)	1957 (44)	1700 (44)	1447 (43)
Female	4517 (55)	4288 (55)	3750 (55)	3321 (55)	3148 (56)	2864 (56)	2500 (56)	2157 (56)	1896 (57)
Education									
High	2070 (25)	1973 (25)	1809 (27)	1645 (27)	1612 (29)	1509 (29)	1355 (30)	1216 (32)	1084 (32)
Medium	3073 (37)	2939 (37)	2557 (38)	2283 (38)	2176 (38)	1982 (39)	1775 (40)	1556 (40)	1375 (41)
Low	3125 (38)	2929 (37)	2435 (36)	2060 (34)	1865 (33)	1646 (32)	1327 (30)	1085 (28)	884 (26)
Wealth									
High	2755 (33)	2637 (34)	2369 (35)	2170 (36)	2081 (37)	1953 (38)	1751 (39)	1534 (40)	1378 (41)
Medium	2756 (33)	2627 (34)	2277 (33)	1996 (33)	1934 (34)	1737 (34)	1525 (34)	1332 (35)	1153 (34)
Low	2757 (33)	2577 (33)	2155 (32)	1822 (30)	1638 (29)	1447 (28)	1181 (26)	991 (26)	812 (24)
Limiting long-standing illness									
No	5720 (69)	5412 (69)	4750 (70)	4275 (71)	4082 (72)	3776 (74)	3317 (74)	2908 (75)	2536 (76)
Yes	2548 (31)	2429 (31)	2051 (30)	1713 (29)	1571 (28)	1361 (26)	1140 (26)	949 (25)	807 (24)
Self-rated health									
Good or better	6417 (78)	6081 (78)	5354 (79)	4807 (80)	4575 (81)	4219 (82)	3712 (83)	3240 (84)	2847 (85)
Fair or worse	1851 (22)	1760 (22)	1447 (21)	1181 (20)	1078 (19)	918 (18)	745 (17)	617 (16)	496 (15)
Smoking									
Nonsmoker	5212 (63)	4942 (63)	4247 (62)	3706 (62)	3472 (61)	3135 (61)	2702 (61)	2296 (60)	1962 (59)
Current	3056 (37)	2899 (37)	2554 (38)	2282 (38)	2181 (39)	2002 (39)	1755 (39)	1561 (40)	1381 (41)
Alcohol consumption									
Less than daily	6486 (78)	6141 (78)	5304 (78)	4646 (78)	4367 (77)	3949 (77)	3413 (77)	2948 (76)	2534 (76)
Daily	1782 (22)	1700 (22)	1497 (22)	1342 (22)	1286 (23)	1188 (23)	1044 (23)	909 (24)	809 (24)
Physical activity									
Moderate or above	5959 (72)	5655 (72)	4983 (73)	4506 (75)	4305 (76)	3983 (78)	3491 (78)	3073 (80)	2690 (80)
Mild or below	2309 (28)	2186 (28)	1818 (27)	1482 (25)	1348 (24)	1154 (22)	966 (22)	784 (20)	653 (20)

Abbreviation: ELSA, English Longitudinal Study of Aging.

In the first outcome, participants experienced a nonlinear decline in memory every 2 years (β linear slope 1, 0.180; SE, 0.022; *P* < .001; and β quadratic slope 1, −0.055; SE, 0.003; *P* < .001) (Table 2). Depressive symptoms were negatively associated with memory at the study baseline (β intercept, −0.018; SE, 0.004; *P* < .001) and with the linear slope of memory change (β linear, −0.146; SE, 0.023; *P* < .001). This suggests that higher levels of depressive symptoms were associated with lower memory scores at the study baseline and a steeper memory decline over time. There were no evident linear or nonlinear changes in verbal fluency (Table 2). Depressive symptoms were negatively associated with verbal fluency at the study baseline (β intercept, −0.009; SE, 0.004; *P* = .02) but not significantly associated with linear slope or quadratic slope of change in verbal fluency scores. This provides evidence for a cross-sectional association but not for a prospective association of higher levels of depressive symptoms at the study baseline with poorer verbal fluency. Investigating the parallel change in cognition and depressive symptoms, the linear slope of change in depressive symptoms was significantly associated with the nonlinear pattern of change in memory for the quadratic slope (β quadratic, −0.253; SE, 0.079; *P* = .001) but not verbal fluency over time (Table 2).

**Table 2.** Bivariate Dual Change Score Model With Bidirectional Coupling Parameters, Outcome Cognition (Memory and Verbal Fluency) (n = 8268)

Factor	Exposure: depressive symptoms			
	Outcome: memory		Outcome: verbal fluency	
	β (SE)	*P* value	β (SE)	*P* value
Baseline cognition (intercept i1)	10.595 (0.081)	<.001	22.401 (0.182)	<.001
Baseline depressive symptoms	−0.018 (0.004)	<.001	−0.009 (0.004)	.02
Baseline age	−0.119 (0.004)	<.001	−0.152 (0.007)	<.001
Sex (female vs male)	0.885 (0.057)	<.001	−0.110 (0.119)	.35
Education				
Medium vs high education	−0.659 (0.070)	<.001	−1.667 (0.160)	<.001
Low vs high education	−1.696 (0.079)	<.001	−3.224 (0.170)	<.001
Wealth				
Medium vs high-wealth	−0.358 (0.067)	<.001	−0.551 (0.146)	<.001
Low vs high-wealth	−0.771 (0.075)	<.001	−1.206 (0.155)	<.001
Limiting long-standing illness	−0.022 (0.070)	.75	−0.093 (0.145)	.52
Self-rated health	−0.452 (0.080)	<.001	−0.681 (0.166)	<.001
Smoking (current vs not current)	0.035 (0.058)	.55	0.263 (0.120)	.03
Alcohol (daily vs less)	0.407 (0.067)	<.001	0.607 (0.145)	<.001
Physical activity (moderate or above vs mild or below)	−0.238 (0.069)	<.001	−0.654 (0.138)	<.001
**The rate of change in cognition**				
Linear slope of cognition (s1)	0.180 (0.022)	<.001	0.094 (0.082)	.250
Baseline depressive symptoms	−0.146 (0.023)	<.001	−0.142 (0.206)	.489
Baseline age	−0.015 (0.002)	<.001	−0.027 (0.009)	.003
Sex (female vs male)	0.046 (0.011)	<.001	0.076 (0.048)	.111
Education				
Medium vs high education	0.008 (0.013)	.51	0.012 (0.049)	.81
Low vs high education	0.006 (0.015)	.68	−0.026 (0.060)	.66
Wealth				
Medium vs high wealth	−0.012 (0.013)	.32	−0.018 (0.058)	.76
Low vs high wealth	−0.023 (0.016)	.15	−0.009 (0.089)	.92
Limiting long-standing illness	0.012 (0.015)	.43	0.008 (0.092)	.93
Self-rated health	0.010 (0.019)	.60	−0.001 (0.139)	.99
Smoking (current vs not current)	−0.012 (0.011)	.26	−0.086 (0.040)	.03
Alcohol (daily vs less)	−0.004 (0.012)	.78	0.036 (0.047)	.44
Physical activity (moderate or above vs mild or below)	−0.008 (0.015)	.59	−0.065 (0.074)	.37
Quadratic slope of cognition (q1)	−0.055 (0.003)	<.001	−0.051 (0.018)	.003
Linear change in depressive symptoms	−0.253 (0.079)	.001	0.001 (0.002)	.71
Variance^a^				
In initial status (i1)	3.914 (0.093)	<.001	16.167 (0.404)	<.001
In the linear rate of change (s1)	0.043 (0.011)	<.001	0.497 (0.131)	<.001
In the quadratic rate of change (q1)	0.001 (0.001)	.001	0.002 (0.011)	.001
Goodness of fit				
RMSEA (90% CI)	0.018 (0.017 to 0.019)		0.015 (0.013 to 0.017)	
AIC	322048.818		263077.115	
BIC	322610.430		263582.565	

Abbreviations: AIC, Akaike information criterion; BIC, bayesian information criterion; RMSEA, root mean square error of approximation.

^a^ The within-person variance is the overall residual variance in cognition (memory or verbal fluency) that is not explained by the model. The initial status variance component is the variance of individual’s intercepts about the intercept of the average person. Likewise, the rate of change variance component is the variance of individual slopes about the slope of the average person.

Exploring the association between memory at the study baseline and depressive symptoms trajectory (Table 3), the participants in this study had a score of depressive symptoms of 0.468 (SE, 0.048; *P* < .001) at the study baseline, a linear slope of β linear slope 2 of 0.002 (SE, 0.010; *P* = .84), and a quadratic slope of β quadratic slope 2 of 0.001 (SE, 0.001; *P* = .39). Memory was found to be inversely associated with the levels of depressive symptoms at the study baseline, as previously noted and associated with the linear change in depressive symptoms over time (β linear, −0.001; SE, 0.001; *P* = .03). Furthermore, investigating the dual changes, the linear slope in memory over time was positively associated with the quadratic slope of change in depressive symptoms (β quadratic, 0.016; SE, 0.006; *P* = .005), despite that the change in depressive symptoms itself was not significant in this study.

**Table 3.** Bivariate Dual Change Score Model With Bidirectional Coupling Parameters, Outcome Depressive Symptoms (n = 8268)

Factor	Outcome: depressive symptoms			
	Exposure: memory		Exposure: verbal fluency	
	β (SE)	*P* value	β (SE)	*P* value
Baseline depressive symptoms (intercept i2)	0.468 (0.048)	<.001	0.473 (0.089)	<.001
Baseline memory	−0.018 (0.004)	<.001	NA	NA
Baseline verbal fluency	NA	NA	−0.009 (0.004)	.02
Baseline age	−0.003 (0.001)	<.001	−0.002 (0.001)	.02
Sex (female vs male)	0.182 (0.012)	<.001	0.166 (0.012)	<.001
Education				
Medium vs high education	0.014 (0.014)	.34	0.011 (0.017)	.52
Low vs high education	0.044 (0.018)	.01	0.046 (0.022)	.03
Wealth				
Medium vs high wealth	0.060 (0.014)	<.001	0.065 (0.015)	<.001
Low vs high wealth	0.139 (0.016)	<.001	0.148 (0.017)	<.001
Limiting long-standing illness	0.224 (0.015)	<.001	0.223 (0.016)	<.001
Self-rated health	0.294 (0.017)	<.001	0.310 (0.018)	<.001
Smoking (current vs not current)	0.043 (0.012)	<.001	0.042 (0.013)	.001
Alcohol (daily vs less)	−0.019 (0.014)	.16	−0.020 (0.015)	.18
Physical activity (moderate or above vs mild or below)	0.101 (0.015)	<.001	0.108 (0.015)	<.001
**The rate of change in depressive symptoms**				
Linear slope of depressive symptoms (s2)	0.002 (0.010)	.84	−0.031 (0.021)	.15
Baseline memory	−0.001 (0.001)	.03	NA	NA
Baseline verbal fluency			−0.451 (0.999)	.65
Baseline age	0.004 (0.001)	<.001	0.003 (0.001)	.005
Sex (female vs male)	−0.001 (0.002)	.76	−0.002 (0.005)	.75
Education				
Medium vs high education	0.001 (0.003)	.74	0.001 (0.006)	.83
Low vs high education	−0.001 (0.003)	.77	0.001 (0.007)	.99
Wealth				
Medium vs high wealth	0.001 (0.003)	.68	−0.002 (0.005)	.70
Low vs high wealth	−0.004 (0.003)	.18	−0.008 (0.006)	.16
Limiting long-standing illness	−0.005 (0.003)	.09	−0.006 (0.005)	.31
Self-rated health	−0.001 (0.004)	.83	−0.012 (0.006)	.05
Smoking (current vs not current)	0.005 (0.002)	.05	0.006 (0.006)	.35
Alcohol (daily vs less)	−0.001 (0.003)	.70	−0.003 (0.005)	.52
Physical activity (moderate or above vs mild or below)	−0.005 (0.003)	.11	−0.010 (0.005)	.06
Quadratic slope of depressive symptoms (q2)	0.001 (0.001)	.39	0.004 (0.002)	.02
Linear change in memory	0.016 (0.006)	.005	NA	NA
Linear change in verbal fluency	NA	NA	0.004 (0.012)	.72
Variance^a^				
In initial status (i2)	0.145 (0.005)	<.001	0.144 (0.004)	<.001
In the linear rate of change (s2)	0.001 (0.001)	<.001	0.003 (0.001)	<.001
In the quadratic rate of change (q2)	0.001 (0.001)	.87	0.001 (0.001)	.87
Goodness of fit				
RMSEA (90% CI)	0.018 (0.017 to 0.019)	NA	0.015 (0.013 to 0.017)	NA
AIC	322048.818	NA	263077.115	NA
BIC	322610.430	NA	263582.565	NA

Abbreviations: AIC, Akaike information criterion; BIC, bayesian information criterion; NA, not available; RMSEA, root mean square error of approximation.

^a^ The within-person variance is the overall residual variance in cognition (memory or verbal fluency) that is not explained by the model. The initial status variance component is the variance of individual’s intercepts about the intercept of the average person. Likewise, the rate of change variance component is the variance of individual slopes about the slope of the average person.

**Figure 2.**
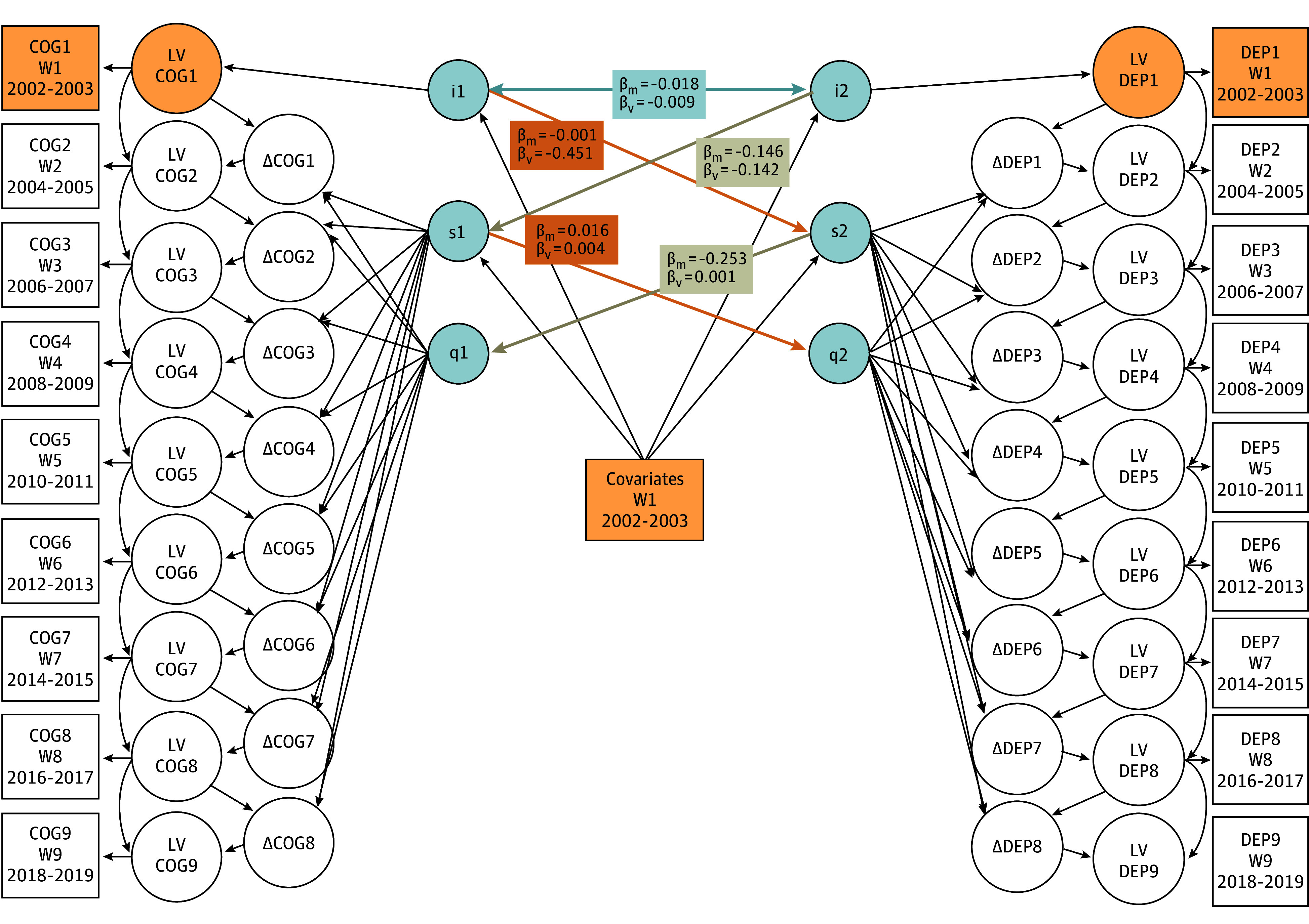
Observed Bidirectional Associations Between Depressive Symptoms and Cognitive Functioning Over Time in ELSA β_m_ indicates β for memory; β_v_, β for verbal fluency; COG, cognitive functioning; DEP, depressive symptoms; ELSA, English Longitudinal Study of Aging; I, intercept; LV, latent variable; Q, quadratic slope; S, linear slope; W, wave.

In the model using verbal fluency to estimate depressive symptoms (Table 3), we noticed an inverse cross-sectional association which suggested that higher verbal fluency scores at the study baseline were associated with less depressive symptoms at the study baseline (β, −0.009; SE, 0.004; *P* = .02). However, we did not observe a prospective association between verbal fluency at the study baseline and a change in depressive symptoms over time. The linear slope in verbal fluency was not associated with the quadratic slope of change in depressive symptoms either. The results are summarized in Figure 2.

In our first sensitivity analysis, where the item regarding loneliness in CES-D was excluded, the results were similar to our main results (eTable 1 and eTable 2 in Supplement 1). The second set of sensitivity analyses restricted the analytical sample to individuals whose cognitive function at the study baseline was not in the lowest quintile (n = 4469) (eTable 3 and eTable 4 in Supplement 1). Depressive symptoms were cross-sectionally associated with memory and verbal fluency at the study baseline and associated with faster memory loss over time. However, memory at the study baseline no longer seemed to estimate a change in depressive symptoms over time. The results appeared similar to the main results after applying censoring at diagnoses of dementia or the first occurrence of stroke (eTable 5 and eTable 6 in Supplement 1).

## Discussion

In this cohort study analyzing a nationally representative sample of English adults aged 50 years or older, we found evidence for a bidirectional association between memory and depressive symptoms over a 16-year period, but the results for verbal fluency remain inconclusive. Baseline depressive symptoms were shown to be cross-sectionally associated with poorer baseline memory and verbal fluency. Baseline depressive symptoms were also associated with a faster decline in memory but not with verbal fluency throughout the follow-up period. Baseline memory function was significantly associated with a linear increase in depressive function. The linear change in depressive symptoms was independently associated with a faster decline in memory but not verbal fluency. In contrast, the rate of decline in memory was associated with an accelerated change in depressive symptoms, but the bidirectional association with verbal fluency remains unclear.

We found that higher depressive symptoms at baseline were associated with poorer baseline memory and faster memory loss over time, which was consistent with previous studies.^24,25,26^ Poorer baseline memory function was associated with subsequent linear increases in depressive symptoms. When examining the dual changes, the rate of change in depressive symptoms was associated with faster memory decline while the linear rate of memory change was associated with greater changes in depressive symptoms. A previous study^27^ found an association with baseline cognitive impairment and a higher subsequent risk of depressive symptoms in an older US population but did not account for dual changes. The association may be only significant when making the comparison between those who are cognitively impaired and those who are not. However, we cannot rule out the possibility that dual changes in depressive symptoms and cognitive function confounded the observed association.

Biologically, depression has been found to negatively affect cognition via increased cortisol levels resulting from dysregulation of the hypothalamic-pituitary–adrenal (HPA) axis.^28,29,30^ This theory is usually referred to as neurotoxicity. Depressive symptoms were also found to be a risk factor for many other health outcomes, including changes in vascular and metabolic systems, glucocorticoid levels, chronic inflammation, and immune system impairment.^28^ These autonomic changes and dysfunction of the HPA axis are part of cumulative responses of human bodies to external stress stimuli, which is usually referred to as allostatic load.^31,32^ When the stimuli are within certain limits, these reactions are beneficial and adaptive. They can be considered part of daily routine to get used to the new external environment or satisfy other essential demands. However, if additional stimuli are superimposed and become chronic, allostatic overload occurs, and the reactions of human bodies can lead to long-term dysregulation in multiple physiological systems. These changes ultimately result in accelerated neurodegeneration, contributing to cognitive dysfunction.^33^ Moreover, having higher depressive symptoms is associated with reduced capability of self-regulation, which leads to a greater chance of unhealthy behaviors that have been shown as risk factors for cognitive impairment.^33^

On the other hand, verbal fluency was only cross-sectionally associated with depressive symptoms at baseline. There was no evidence suggesting baseline depressive symptoms estimated cognitive change or baseline cognition estimated changes in depressive symptoms. Multiple brain regions and various brain processes are involved in performance on verbal fluency tests. Frontal lesions^34,35^ and temporal lesions^36,37^ have been shown in individuals with impaired fluency. Successful performance on category fluency tasks is affected by both executive control ability and verbal ability.^38^ Although function in some cognitive domains, like memory, shows a substantial decrease, this is different for other cognitive aspects, such as verbal fluency.^39^ Mental abilities, like vocabulary or calculation, are shown to decline at a much slower rate compared with abilities like reasoning skills.^40^ Furthermore, in our study, verbal fluency results were only available from waves 1 to 5 and showed little change over this period. If we had data at later waves, it may be possible to observe significant differences in verbal fluency function.

Depressive symptoms have been shown to be closely associated with loneliness,^41,42^^,^ which was found to be bidirectionally associated with cognitive decline.^13^ They do overlap substantially but are indeed distinct constructs.^42^ The association between depressive symptoms and cognitive function seen in this study was not only independent of loneliness but also different from the associations between loneliness and cognition observed in the same population.^13^ This provides additional evidence for the distinction between feelings of loneliness and depressive symptoms and suggests that they might have different impacts on cognitive functioning.

Our study has several strengths. We benefitted from longitudinal data from a large nationally representative sample of English adults aged 50 years and older throughout a follow-up period of up to 16 years. The statistical model used was powerful enough to examine the directionality of the association of depressive symptoms with memory and verbal fluency.^43^ To our knowledge, this is the first study to explore the dual parallel changes in depressive symptoms and cognitive function.

Based on the bidirectional association between depressive symptoms and memory, these findings suggest that individuals presenting with depressive symptoms should be assessed for potential memory deficits over time. Clinicians should consider regular memory assessment as part of the evaluation for depressive symptoms. Addressing memory issues in individuals with depression may be important for both monitoring cognitive function and improving psychological well-being. Similarly, addressing depressive symptoms in individuals with memory loss may help mitigate further decline in memory function.

Finally, our extended follow-up period highlights the need for regular and longitudinal monitoring of patients with depressive symptoms or memory loss symptoms. This could allow for early detection of changes and appropriate intervention strategies to be implemented and may slow the progression of memory decline associated with dementia and improve overall psychological well-being. Integrated treatment approaches, including psychotherapy or pharmacotherapy for depression and cognitive interventions for memory deficits, could be considered to alleviate the decline in mental functioning and psychological well-being in middle-aged and older populations.

### Limitations

This study has limitations. As with most longitudinal studies of aging, a substantial number of the core participants were lost to follow-up, primarily due to death. These participants were older, more likely to be men, and less affluent. They had lower educational attainment, limiting long-standing illness, fair or worse overall health, milder physical activity and poorer cognition at baseline (eTable 7 in Supplement 1). This suggests that the observed results may be conservative despite the relatively high attrition throughout the follow-up period. The associations might be more robust if data for participants who gradually dropped out were available. Although we carried out sensitivity analyses in which the loneliness item of the CES-D was excluded, there may be residual confounding due to the impact of loneliness. Appropriate adjustment for feelings of loneliness may be desired in future studies. Furthermore, depressive symptoms were measured at a syndrome level. Different depressive symptoms may represent various domains and have distinct associations with cognitive domains.^44^ The inclusion of measures on different syndromes may help us obtain a better understanding of the underlying association and it could provide an improved understanding of the bidirectional association of depressive symptoms if other cognitive domains were available. Finally, because of the observational nature of this study, the ability to establish clear causality was limited.

## Conclusions

Depressive symptoms appeared to be associated with poorer memory at baseline and contributed to faster memory loss over time. In reverse, poorer memory seemed to be associated with greater depressive symptoms at baseline and greater change in depressive symptoms over time. However, given the shorter period of follow-up for verbal fluency, our results on the bidirectional association between depressive symptoms and verbal fluency remain inconclusive. In summary, these findings highlight the complex interplay between depressive symptoms and memory loss, underscoring the importance of integrated assessment and treatment approaches in clinical practice and suggesting that early intervention in depressive symptoms could provide a timely opportunity to slow down or delay memory decline in later life.

## Notes

Supplement 1. **eTable 1.** Bivariate Dual Change Score Model With Bidirectional Coupling Parameters Excluding the Item About Loneliness in CES-D, Outcome Cognition **eTable 2.** Bivariate Dual Change Score Model With Bidirectional Coupling Parameters Excluding the Item About Loneliness in CES-D, Outcome Depressive Symptoms **eTable 3.** Bivariate Dual Change Score Model With Bidirectional Coupling Parameters Excluding Individuals With Baseline Cognition in the Lowest Quintile, Outcome Cognition **eTable 4.** Bivariate Dual Change Score Model With Bidirectional Coupling Parameters Excluding Individuals With Baseline Cognition in the Lowest Quintile, Outcome Depressive Symptoms **eTable 5.** Bivariate Dual Change Score Model With Bidirectional Coupling Parameters Censoring at Diagnoses of Dementia or Stroke, Outcome Cognition **eTable 6.** Bivariate Dual Change Score Model With Bidirectional Coupling Parameters Censoring at Diagnoses of Dementia or Stroke, Outcome Depressive Symptoms **eTable 7.** Baseline Characteristics of Participants With Complete Data and Those Lost to Follow-Up

Supplement 2.
Data Sharing Statement
