# Supplementary material for: Bidirectional Associations of Depressive Symptoms and Cognitive Function Over Time
Source: JAMA Netw Open. 2024 Jun 11;7(6):e2416305. doi: 10.1001/jamanetworkopen.2024.16305 (PMC11167501; doi:10.1001/jamanetworkopen.2024.16305)
Supplement: Supplement 1. — eTable 1. Bivariate Dual Change Score Model With Bidirectional Coupling Parameters Excluding the Item About Loneliness in CES-D, Outcome Cognition eTable 2. Bivariate Dual Change Score Model With Bidirectional Coupling Parameters Excluding the Item About Loneliness in CES-D, Outcome Depressive Symptoms eTable 3. Bivariate Dual Change Score Model With Bidirectional Coupling Parameters Excluding Individuals With Baseline Cognition in the Lowest Quintile, Outcome Cognition eTable 4. Bivariate Dual Change Score Model With Bidirectional Coupling Parameters Excluding Individuals With Baseline Cognition in the Lowest Quintile, Outcome Depressive Symptoms eTable 5. Bivariate Dual Change Score Model With Bidirectional Coupling Parameters Censoring at Diagnoses of Dementia or Stroke, Outcome Cognition eTable 6. Bivariate Dual Change Score Model With Bidirectional Coupling Parameters Censoring at Diagnoses of Dementia or Stroke, Outcome Depressive Symptoms eTable 7. Baseline Characteristics of Participants With Complete Data and Those Lost to Follow-Up [file jamanetwopen-e2416305-s001.pdf]

Supplemental Online Content

Yin J, John A, Cadar D. Bidirectional association of depressive symptoms and cognitive function over time. *JAMA Netw Open*. 2024;7(6):e2416305. doi:10.1001/jamanetworkopen.2024.16305

eTable 1. Bivariate Dual Change Score Model With Bidirectional Coupling Parameters Excluding the Item About Loneliness in CES-D, Outcome Cognition

eTable 2. Bivariate Dual Change Score Model With Bidirectional Coupling Parameters Excluding the Item About Loneliness in CES-D, Outcome Depressive Symptoms

eTable 3. Bivariate Dual Change Score Model With Bidirectional Coupling Parameters Excluding Individuals With Baseline Cognition in the Lowest Quintile, Outcome Cognition

eTable 4. Bivariate Dual Change Score Model With Bidirectional Coupling Parameters Excluding Individuals With Baseline Cognition in the Lowest Quintile, Outcome Depressive Symptoms

eTable 5. Bivariate Dual Change Score Model With Bidirectional Coupling Parameters Censoring at Diagnoses of Dementia or Stroke, Outcome Cognition

eTable 6. Bivariate Dual Change Score Model With Bidirectional Coupling Parameters Censoring at Diagnoses of Dementia or Stroke, Outcome Depressive Symptoms

eTable 7. Baseline Characteristics of Participants With Complete Data and Those Lost to Follow-Up

This supplemental material has been provided by the authors to give readers additional information about their work.

© 2024 Yin J et al. *JAMA Network Open.*

eTable 1 Bivariate dual change score model with bidirectional coupling parameters excluding the item about loneliness in CES-D, outcome cognition (memory and verbal fluency) (n=8,268)

	Outcome: Memory		Outcome: Verbal fluency	
	Exposure: depressive symptoms			
Initial status: cognition	β (SE)	P-value	β (SE)	P-value
Baseline cognition (intercept i1)	10.594 (0.081)	0.001	22.395 (0.184)	0.001
Baseline depressive symptoms	-0.017 (0.005)	0.001	-0.601 (1.609)	0.71
Baseline age	-0.118 (0.003)	0.001	-0.153 (0.008)	0.001
Sex (female vs male)	0.882 (0.057)	0.001	-0.112 (0.119)	0.35
Education				
Medium vs high education	-0.658 (0.071)	0.001	-1.670 (0.162)	0.001
Low vs high education	-1.695 (0.079)	0.001	-3.226 (0.170)	0.001
Wealth				
Medium vs high-wealth	-0.357 (0.067)	0.001	-0.545 (0.147)	0.001
Low vs high-wealth	-0.767 (0.075)	0.001	-1.201 (0.158)	0.001
Limiting long-standing illness	-0.021 (0.070)	0.76	-0.088 (0.148)	0.56
Self-rated health	-0.452 (0.080)	0.001	-0.669 (0.180)	0.001
Smoking (current vs. not current)	0.034 (0.058)	0.56	0.264 (0.120)	0.03
Alcohol (daily vs less)	0.408 (0.067)	0.001	0.609 (0.145)	0.001
Physical activity (moderate or above vs mild or below)	-0.236 (0.069)	0.001	-0.656 (0.139)	0.001
The rate of change in cognition				
Linear slope of cognition (s1)	0.176 (0.023)	0.001	0.088 (0.084)	0.29
Baseline depressive symptoms	-0.139 (0.033)	0.001	-0.090 (0.310)	0.77
Baseline age	-0.015 (0.002)	0.001	-0.026 (0.012)	0.02
Sex (female vs male)	0.045 (0.012)	0.001	0.068 (0.056)	0.23
Education				
Medium vs high education	0.008 (0.013)	0.56	0.015 (0.053)	0.77
Low vs high education	0.004 (0.015)	0.79	-0.027 (0.060)	0.65
Wealth				
Medium vs high wealth	-0.014 (0.013)	0.28	-0.028 (0.076)	0.71
Low vs high wealth	-0.030 (0.018)	0.10	-0.026 (0.126)	0.83
Limiting long-standing illness	0.009 (0.017)	0.61	-0.011 (0.131)	0.94
Self-rated health	0.006 (0.022)	0.78	-0.037 (0.232)	0.87
Smoking (current vs. not current)	-0.012 (0.011)	0.28	-0.090 (0.040)	0.02
Alcohol (daily vs less)	-0.003 (0.013)	0.81	0.037 (0.047)	0.43
Physical activity (moderate or above vs mild or below)	-0.011 (0.016)	0.48	-0.074 (0.102)	0.46
Quadratic slope of cognition (q1)	-0.056 (0.003)	0.001	-0.054 (0.023)	0.02
Linear change in depressive symptoms	-0.280 (0.097)	0.004	-0.601 (1.609)	0.71
Variance				
In initial status (i1)	3.906 (0.092)	0.001	16.194 (0.468)	0.001
In the linear rate of change (s1)	0.049 (0.011)	0.001	0.511 (0.129)	0.001
In the quadratic rate of change (q1)	0.001 (0.001)	0.005	0.001 (0.012)	0.98
Goodness of fit				
RMSEA (90% CI)	0.017 (0.016, 0.018)		0.015 (0.013, 0.017)	
AIC	319307.653		261301.526	
BIC	319869.265		261806.976	

© 2024 Yin J et al. *JAMA Network Open.*

β, beta coefficient; SE, standard error; RMSEA, root mean square error of approximation; AIC, Akaike's Information Criterion; BIC, Bayesian Information Criterion; 95% CI, confidence intervals.
aThe within-person variance is the overall residual variance in cognition (memory or verbal fluency) that is not explained by the model. The initial status variance component is the variance of individual's intercepts about the intercept of the average person. Likewise, the rate of change variance component is the variance of individual slopes about the slope of the average person.

© 2024 Yin J et al. *JAMA Network Open.*

eTable 2 Bivariate dual change score model with bidirectional coupling parameters excluding the item about loneliness in CES-D, outcome depressive symptoms (n=11,872)

	Outcome: Depressive symptoms			
	Exposure: Memory		Exposure: Verbal fluency	
Initial status: Depressive Symptoms	β (SE)	P-value	β (SE)	P-value
Baseline depressive symptoms (intercept i2)	0.445 (0.051)	0.001	0.448 (0.105)	0.001
Baseline memory	-0.017 (0.005)	0.001		
Baseline verbal fluency			-0.009 (0.005)	0.07
Baseline age	-0.004 (0.001)	0.001	-0.002 (0.001)	0.001
Sex (female vs male)	0.169 (0.012)	0.001	0.156 (0.012)	0.001
Education				
Medium vs high education	0.015 (0.014)	0.29	0.009 (0.017)	0.58
Low vs high education	0.038 (0.017)	0.03	0.037 (0.023)	0.10
Wealth				
Medium vs high wealth	0.053 (0.013)	0.001	0.060 (0.014)	0.001
Low vs high wealth	0.121 (0.015)	0.001	0.130 (0.016)	0.001
Limiting long-standing illness	0.218 (0.014)	0.001	0.217 (0.015)	0.001
Self-rated health	0.288 (0.017)	0.001	0.308 (0.017)	0.001
Smoking (current vs. not current)	0.041 (0.011)	0.001	0.041 (0.013)	0.001
Alcohol (daily vs less)	-0.020 (0.013)	0.14	-0.021 (0.014)	0.13
Physical activity (moderate or above vs mild or below)	0.096 (0.014)	0.001	0.103 (0.015)	0.001
The rate of change in depressive symptoms				
Linear slope of depressive symptoms (s2)	-0.005 (0.011)	0.64	-0.015 (0.045)	0.73
Baseline memory	0.001 (0.001)	0.04		
Baseline verbal fluency			0.001 (0.002)	0.73
Baseline age	0.003 (0.001)	0.001	0.003 (0.001)	0.05
Sex (female vs male)	-0.001 (0.002)	0.81	-0.001 (0.005)	0.79
Education				
Medium vs high education	0.001 (0.003)	0.68	0.003 (0.007)	0.69
Low vs high education	0.001 (0.003)	0.95	0.003 (0.008)	0.75
Wealth				
Medium vs high wealth	0.001 (0.003)	0.59	-0.003 (0.006)	0.62
Low vs high wealth	-0.004 (0.003)	0.16	-0.008 (0.005)	0.12
Limiting long-standing illness	-0.005 (0.003)	0.07	-0.006 (0.006)	0.34
Self-rated health	-0.001 (0.003)	0.71	-0.015 (0.006)	0.02
Smoking (current vs. not current)	0.004 (0.002)	0.06	0.004 (0.007)	0.62
Alcohol (daily vs less)	0.001 (0.003)	0.86	-0.002 (0.005)	0.76
Physical activity (moderate or above vs mild or below)	-0.005 (0.003)	0.08	-0.010 (0.005)	0.08
Quadratic slope of depressive symptoms (q2)	0.002 (0.001)	0.09	0.004 (0.002)	0.02
Linear change in memory	0.012 (0.005)	0.02		
Linear change in verbal fluency			0.001 (0.015)	0.94
Variance				
In initial status (i2)	0.128 (0.004)	0.001	0.127 (0.004)	0.001
In the linear rate of change (s2)	0.001 (0.001)	0.005	0.003 (0.001)	0.01
In the quadratic rate of change (q2)	0.001 (0.001)	0.49	0.001 (0.001)	0.83
Goodness of fit				

RMSEA (90% CI)	0.017 (0.016, 0.018)	0.015 (0.013, 0.017)
AIC	319307.653	261301.526
BIC	319869.265	261806.976

β, beta coefficient; SE, standard error; RMSEA, root mean square error of approximation; AIC, Akaike's Information Criterion; BIC, Bayesian Information Criterion; 95% CI, confidence intervals.

aThe within-person variance is the overall residual variance in cognition (memory or verbal fluency) that is not explained by the model. The initial status variance component is the variance of individual's intercepts about the intercept of the average person. Likewise, the rate of change variance component is the variance of individual slopes about the slope of the average person.

© 2024 Yin J et al. *JAMA Network Open.*

eTable 3 Bivariate dual change score model with bidirectional coupling parameters excluding individuals with baseline cognition in the lowest quintile, outcome cognition (memory and verbal fluency) (n=4,469)

	Outcome: Memory		Outcome: Verbal fluency	
	Exposure: depressive symptoms			
Initial status: cognition	β (SE)	P-value	β (SE)	P-value
Baseline cognition (intercept i1)	11.879 (0.081)	0.001	22.401 (0.182)	0.001
Baseline depressive symptoms	-0.017 (0.009)	0.04	-0.009 (0.004)	0.02
Baseline age	-0.052 (0.004)	0.001	-0.152 (0.007)	0.001
Sex (female vs male)	0.619 (0.058)	0.001	-0.110 (0.119)	0.35
Education				
Medium vs high education	-0.491 (0.069)	0.001	-1.667 (0.160)	0.001
Low vs high education	-1.086 (0.078)	0.001	-3.224 (0.170)	0.001
Wealth				
Medium vs high-wealth	-0.248 (0.067)	0.001	-0.551 (0.146)	0.001
Low vs high-wealth	-0.356 (0.076)	0.001	-1.206 (0.155)	0.001
Limiting long-standing illness	-0.085 (0.071)	0.23	-0.093 (0.145)	0.52
Self-rated health	-0.164 (0.085)	0.05	-0.681 (0.166)	0.001
Smoking (current vs. not current)	0.043 (0.060)	0.48	0.263 (0.120)	0.03
Alcohol (daily vs less)	0.221 (0.067)	0.001	0.607 (0.145)	0.001
Physical activity (moderate or above vs mild or below)	-0.007 (0.072)	0.93	-0.654 (0.138)	0.001
The rate of change in cognition				
Linear slope of cognition (s1)	-0.145 (0.030)	0.001	0.094 (0.082)	0.25
Baseline depressive symptoms	-0.102 (0.036)	0.005	-0.142 (0.206)	0.49
Baseline age	-0.028 (0.003)	0.001	-0.027 (0.009)	0.003
Sex (female vs male)	0.073 (0.017)	0.001	0.076 (0.048)	0.11
Education				
Medium vs high education	-0.013 (0.018)	0.47	0.012 (0.049)	0.81
Low vs high education	-0.058 (0.023)	0.01	-0.026 (0.060)	0.66
Wealth				
Medium vs high wealth	-0.007 (0.018)	0.69	-0.018 (0.058)	0.76
Low vs high wealth	-0.054 (0.022)	0.02	-0.009 (0.089)	0.92
Limiting long-standing illness	0.007 (0.022)	0.77	0.008 (0.092)	0.93
Self-rated health	-0.042 (0.026)	0.12	-0.001 (0.139)	0.99
Smoking (current vs. not current)	-0.023 (0.016)	0.14	-0.086 (0.040)	0.03
Alcohol (daily vs less)	0.007 (0.018)	0.69	0.036 (0.047)	0.44
Physical activity (moderate or above vs mild or below)	-0.016 (0.025)	0.52	-0.065 (0.074)	0.37
Quadratic slope of cognition (q1)	-0.021 (0.004)	0.001	-0.051 (0.018)	0.003
Linear change in depressive symptoms	0.086 (0.149)	0.56	-0.451 (0.999)	0.65
Variance				
In initial status (i1)	1.929 (0.070)	0.001	16.167 (0.404)	0.001
In the linear rate of change (s1)	0.093 (0.008)	0.001	0.497 (0.131)	0.001
In the quadratic rate of change (q1)	0.001 (0.001)	0.43	0.002 (0.011)	0.88
Goodness of fit				
RMSEA (90% CI)	0.021 (0.020, 0.023)		0.015 (0.013, 0.017)	
AIC	186135.305		263077.115	
BIC	186647.699		263582.565	

© 2024 Yin J et al. *JAMA Network Open.*

β, beta coefficient; SE, standard error; RMSEA, root mean square error of approximation; AIC, Akaike's Information Criterion; BIC, Bayesian Information Criterion; 95% CI, confidence intervals.
aThe within-person variance is the overall residual variance in cognition (memory or verbal fluency) that is not explained by the model. The initial status variance component is the variance of individual's intercepts about the intercept of the average person. Likewise, the rate of change variance component is the variance of individual slopes about the slope of the average person.

© 2024 Yin J et al. *JAMA Network Open.*

eTable 4 Bivariate dual change score model with bidirectional coupling parameters excluding individuals with baseline cognition in the lowest quintile, outcome depressive symptoms (n=4,469)

	Outcome: Depressive symptoms			
	Exposure: Memory		Exposure: Verbal fluency	
Initial status: Depressive Symptoms	β (SE)	P-value	β (SE)	P-value
Baseline depressive symptoms (intercept i2)	0.463 (0.105)	0.001	0.473 (0.089)	0.001
Baseline memory	-0.017 (0.009)	0.04		
Baseline verbal fluency			-0.009 (0.004)	0.02
Baseline age	-0.003 (0.001)	0.005	-0.002 (0.001)	0.02
Sex (female vs male)	0.170 (0.016)	0.001	0.166 (0.012)	0.001
Education				
Medium vs high education	0.022 (0.018)	0.21	0.011 (0.017)	0.52
Low vs high education	0.045 (0.024)	0.06	0.046 (0.022)	0.03
Wealth				
Medium vs high wealth	0.077 (0.017)	0.001	0.065 (0.015)	0.001
Low vs high wealth	0.114 (0.021)	0.001	0.148 (0.017)	0.001
Limiting long-standing illness	0.225 (0.020)	0.001	0.223 (0.016)	0.001
Self-rated health	0.279 (0.026)	0.001	0.310 (0.018)	0.001
Smoking (current vs. not current)	0.046 (0.016)	0.003	0.042 (0.013)	0.001
Alcohol (daily vs less)	-0.004 (0.017)	0.80	-0.020 (0.015)	0.18
Physical activity (moderate or above vs mild or below)	0.085 (0.021)	0.001	0.108 (0.015)	0.001
The rate of change in depressive symptoms				
Linear slope of depressive symptoms (s2)	0.015 (0.021)	0.47	-0.012 (0.037)	0.75
Baseline memory	-0.002 (0.002)	0.37		
Baseline verbal fluency			0.001 (0.002)	0.71
Baseline age	0.003 (0.001)	0.001	0.003 (0.001)	0.005
Sex (female vs male)	0.002 (0.003)	0.56	-0.002 (0.005)	0.75
Education				
Medium vs high education	0.002 (0.003)	0.58	0.001 (0.006)	0.83
Low vs high education	0.002 (0.005)	0.69	0.001 (0.007)	0.99
Wealth				
Medium vs high wealth	-0.003 (0.003)	0.31	-0.002 (0.005)	0.70
Low vs high wealth	-0.002 (0.004)	0.67	-0.008 (0.006)	0.16
Limiting long-standing illness	-0.004 (0.004)	0.28	-0.006 (0.005)	0.31
Self-rated health	0.002 (0.005)	0.70	-0.012 (0.006)	0.05
Smoking (current vs. not current)	0.002 (0.003)	0.52	0.006 (0.006)	0.35
Alcohol (daily vs less)	-0.003 (0.003)	0.39	-0.003 (0.005)	0.52
Physical activity (moderate or above vs mild or below)	-0.009 (0.004)	0.03	-0.010 (0.005)	0.06
Quadratic slope of depressive symptoms (q2)	0.003 (0.001)	0.001	0.004 (0.002)	0.02
Linear change in memory	0.004 (0.002)	0.07		
Linear change in verbal fluency			0.004 (0.012)	0.72
Variance				
In initial status (i2)	0.129 (0.004)	0.001	0.144 (0.004)	0.001
In the linear rate of change (s2)	0.001 (0.001)	0.04	0.003 (0.001)	0.02
In the quadratic rate of change (q2)	0.001 (0.001)	0.008	0.001 (0.001)	0.82
Goodness of fit				

© 2024 Yin J et al. *JAMA Network Open.*

RMSEA (90% CI)	0.021 (0.020, 0.023)	0.015 (0.013, 0.017)
AIC	186135.305	263077.115
BIC	186647.699	263582.565

β, beta coefficient; SE, standard error; RMSEA, root mean square error of approximation; AIC, Akaike's Information Criterion; BIC, Bayesian Information Criterion; 95% CI, confidence intervals.
aThe within-person variance is the overall residual variance in cognition (memory or verbal fluency) that is not explained by the model. The initial status variance component is the variance of individual's intercepts about the intercept of the average person. Likewise, the rate of change variance component is the variance of individual slopes about the slope of the average person.

© 2024 Yin J et al. *JAMA Network Open.*

eTable 5 Bivariate dual change score model with bidirectional coupling parameters censoring at diagnoses of dementia or stroke, outcome cognition (n=10,501)

	Outcome: Memory		Outcome: Verbal fluency	
	Exposure: depressive symptoms			
Initial status: cognition	β (SE)	P-value	β (SE)	P-value
Baseline cognition (intercept i1)	10.595 (0.081)	0.001	22.401 (0.182)	0.001
Baseline depressive symptoms	-0.018 (0.004)	0.001	-0.009 (0.004)	0.02
Baseline age	-0.119 (0.004)	0.001	-0.152 (0.007)	0.001
Sex (female vs male)	0.885 (0.057)	0.001	-0.110 (0.119)	0.35
Education				
Medium vs high education	-0.659 (0.070)	0.001	-1.667 (0.160)	0.001
Low vs high education	-1.696 (0.079)	0.001	-3.224 (0.170)	0.001
Wealth				
Medium vs high-wealth	-0.358 (0.067)	0.001	-0.551 (0.146)	0.001
Low vs high-wealth	-0.771 (0.075)	0.001	-1.206 (0.155)	0.001
Limiting long-standing illness	-0.022 (0.070)	0.75	-0.093 (0.145)	0.521
Self-rated health	-0.452 (0.080)	0.001	-0.681 (0.166)	0.001
Smoking (current vs. not current)	0.035 (0.058)	0.55	0.263 (0.120)	0.03
Alcohol (daily vs less)	0.407 (0.067)	0.001	0.607 (0.145)	0.001
Physical activity (moderate or above vs mild or below)	-0.238 (0.069)	0.001	-0.654 (0.138)	0.001
The rate of change in cognition				
Linear slope of cognition (s1)	0.180 (0.022)	0.001	0.094 (0.082)	0.25
Baseline depressive symptoms	-0.146 (0.023)	0.001	-0.142 (0.206)	0.49
Baseline age	-0.015 (0.002)	0.001	-0.027 (0.009)	0.003
Sex (female vs male)	0.046 (0.011)	0.001	0.076 (0.048)	0.11
Education				
Medium vs high education	0.008 (0.013)	0.51	0.012 (0.049)	0.81
Low vs high education	0.006 (0.015)	0.68	-0.026 (0.060)	0.66
Wealth				
Medium vs high wealth	-0.012 (0.013)	0.32	-0.018 (0.058)	0.76
Low vs high wealth	-0.023 (0.016)	0.15	-0.009 (0.089)	0.92
Limiting long-standing illness	0.012 (0.015)	0.43	0.008 (0.092)	0.93
Self-rated health	0.010 (0.019)	0.60	-0.001 (0.139)	0.99
Smoking (current vs. not current)	-0.012 (0.011)	0.26	-0.086 (0.040)	0.03
Alcohol (daily vs less)	-0.004 (0.012)	0.78	0.036 (0.047)	0.44
Physical activity (moderate or above vs mild or below)	-0.008 (0.015)	0.59	-0.065 (0.074)	0.37
Quadratic slope of cognition (q1)	-0.055 (0.003)	0.001	-0.051 (0.018)	0.003
Linear change in depressive symptoms	-0.253 (0.079)	0.001	-0.451 (0.999)	0.65
Variance				
In initial status (i1)	3.914 (0.093)	0.001	16.167 (0.404)	0.001
In the linear rate of change (s1)	0.043 (0.011)	0.001	0.497 (0.131)	0.001
In the quadratic rate of change (q1)	0.001 (0.001)	0.001	0.002 (0.011)	0.88
Goodness of fit				
RMSEA (90% CI)	0.018 (0.017, 0.019)		0.015 (0.013, 0.017)	
AIC	322048.818		263077.115	
BIC	322610.430		263582.565	

© 2024 Yin J et al. *JAMA Network Open*.

β, beta coefficient; SE, standard error; RMSEA, root mean square error of approximation; AIC, Akaike's Information Criterion; BIC, Bayesian Information Criterion; 95% CI, confidence intervals.

aThe within-person variance is the overall residual variance in cognition (memory or verbal fluency) that is not explained by the model. The initial status variance component is the variance of individual's intercepts about the intercept of the average person. Likewise, the rate of change variance component is the variance of individual slopes about the slope of the average person.

© 2024 Yin J et al. *JAMA Network Open*.

eTable 6 Bivariate dual change score model with bidirectional coupling parameters censoring at diagnoses of dementia or stroke, outcome depressive symptoms (n=10,501)

	Outcome: Depressive symptoms			
	Exposure: Memory		Exposure: Verbal fluency	
Initial status: Depressive Symptoms	β (SE)	P-value	β (SE)	P-value
Baseline depressive symptoms (intercept i2)	0.468 (0.048)	0.001	0.473 (0.089)	0.001
Baseline memory	-0.018 (0.004)	0.001		
Baseline verbal fluency			-0.009 (0.004)	0.02
Baseline age	-0.003 (0.001)	0.001	-0.002 (0.001)	0.016
Sex (female vs male)	0.182 (0.012)	0.001	0.166 (0.012)	0.001
Education				
Medium vs high education	0.014 (0.014)	0.34	0.011 (0.017)	0.52
Low vs high education	0.044 (0.018)	0.01	0.046 (0.022)	0.03
Wealth				
Medium vs high wealth	0.060 (0.014)	0.001	0.065 (0.015)	0.001
Low vs high wealth	0.139 (0.016)	0.001	0.148 (0.017)	0.001
Limiting long-standing illness	0.224 (0.015)	0.001	0.223 (0.016)	0.001
Self-rated health	0.294 (0.017)	0.001	0.310 (0.018)	0.001
Smoking (current vs. not current)	0.043 (0.012)	0.001	0.042 (0.013)	0.001
Alcohol (daily vs less)	-0.019 (0.014)	0.16	-0.020 (0.015)	0.18
Physical activity (moderate or above vs mild or below)	0.101 (0.015)	0.001	0.108 (0.015)	0.001
The rate of change in depressive symptoms				
Linear slope of depressive symptoms (s2)	0.002 (0.010)	0.84	-0.012 (0.037)	0.75
Baseline memory	-0.001 (0.001)	0.04		
Baseline verbal fluency			0.001 (0.002)	0.71
Baseline age	0.004 (0.001)	0.001	0.003 (0.001)	0.005
Sex (female vs male)	-0.001 (0.002)	0.76	-0.002 (0.005)	0.75
Education				
Medium vs high education	0.001 (0.003)	0.74	0.001 (0.006)	0.83
Low vs high education	-0.001 (0.003)	0.77	0.001 (0.007)	0.99
Wealth				
Medium vs high wealth	0.001 (0.003)	0.68	-0.002 (0.005)	0.70
Low vs high wealth	-0.004 (0.003)	0.18	-0.008 (0.006)	0.16
Limiting long-standing illness	-0.005 (0.003)	0.09	-0.006 (0.005)	0.31
Self-rated health	-0.001 (0.004)	0.83	-0.012 (0.006)	0.05
Smoking (current vs. not current)	0.005 (0.002)	0.04	0.006 (0.006)	0.35
Alcohol (daily vs less)	-0.001 (0.003)	0.70	-0.003 (0.005)	0.52
Physical activity (moderate or above vs mild or below)	-0.005 (0.003)	0.11	-0.010 (0.005)	0.06
Quadratic slope of depressive symptoms (q2)	0.001 (0.001)	0.39	0.004 (0.002)	0.02
Linear change in memory	0.016 (0.006)	0.005		
Linear change in verbal fluency			0.004 (0.012)	0.72
Variance				
In initial status (i2)	0.145 (0.005)	0.001	0.144 (0.004)	0.001
In the linear rate of change (s2)	0.001 (0.001)	0.001	0.003 (0.001)	0.02
In the quadratic rate of change (q2)	0.001 (0.001)	0.87	0.001 (0.001)	0.82
Goodness of fit				

© 2024 Yin J et al. *JAMA Network Open.*

RMSEA (90% CI)	0.018 (0.017, 0.019)	0.015 (0.013, 0.017)
AIC	322048.818	263077.115
BIC	322610.430	263582.565

β, beta coefficient; SE, standard error; RMSEA, root mean square error of approximation; AIC, Akaike's Information Criterion; BIC, Bayesian Information Criterion; 95% CI, confidence intervals.

aThe within-person variance is the overall residual variance in cognition (memory or verbal fluency) that is not explained by the model. The initial status variance component is the variance of individual's intercepts about the intercept of the average person. Likewise, the rate of change variance component is the variance of individual slopes about the slope of the average person.

© 2024 Yin J et al. *JAMA Network Open*.

eTable 7 Baseline characteristics of participants who stayed in the study and lost to follow-up

	Stayed in study (N=3,343) n (%)	Lost to follow-up (N=4,925) n (%)	p-value
Memory			
Mean (SD)	11 (3.0)	9 (3.5)	<0.001
Range	0 to 20	0 to 20	
Verbal fluency			
Mean (SD)	21 (6.2)	19 (6.0)	<0.001
Range	0 to 55	0 to 49	
Depressive symptoms			
Median (IQR)	1 (0, 2)	1 (0, 2)	<0.001
Range	0 to 8	0 to 8	
Age, years			
Mean (SD)	60 (7.1)	67 (10)	<0.001
Sex			
Male	1447 (43)	2304 (47)	0.002
Female	1896 (57)	2621 (53)	
Education			
High	1084 (32)	986 (20)	
Medium	1375 (41)	1698 (34)	<0.001
Low	884 (26)	2241 (46)	
Wealth			
High	1378 (41)	1377 (28)	
Medium	1153 (34)	1603 (33)	<0.001
Low	812 (24)	1945 (39)	
Limiting long-standing illness			
No	2536 (76)	3184 (65)	<0.001
Yes	807 (24)	1741 (35)	
Self-rated health			
Good or better	2847 (85)	3570 (72)	<0.001
Fair or worse	496 (15)	1355 (28)	
Smoking			
Non-smoker	1962 (59)	3250 (66)	<0.001
Current	1381 (41)	1675 (34)	
Alcohol consumption			
Less than daily	2534 (76)	3952 (80)	<0.001
Daily	809 (24)	973 (20)	
Physical activity			
Moderate or above	2690 (80)	3269 (66)	<0.001
Mild or below	653 (20)	1656 (34)	

SD, standard deviation. IQR, interquartile range.

© 2024 Yin J et al. *JAMA Network Open.*
