# Supplementary material for: Bidirectional Associations of Depressive Symptoms and Cognitive Function Over Time
Source: JAMA Netw Open. 2024 Jun 11;7(6):e2416305. doi: 10.1001/jamanetworkopen.2024.16305 (PMC11167501; doi:10.1001/jamanetworkopen.2024.16305)
Supplement: Supplement 2. — Data Sharing Statement [file jamanetwopen-e2416305-s002.pdf]

Data Sharing Statement

Yin. Bidirectional Associations of Depressive Symptoms and Cognitive Function Over Time. *JAMA Netw Open*. Published June 11, 2024. doi:10.1001/jamanetworkopen.2024.16305

Data
Data available: Yes
Data types: Deidentified participant data
How to access data: The English Longitudinal Study of Ageing (ELSA) is an open-access database. The data are available via UK Data Services SN: 200011 (https://www.ukdataservice.ac.uk/datacatalogue). For more information, visit the study website (https://www.elsa-project.ac.uk/accessing-elsa-data).
When available: With publication

Supporting Documents
Document types: None

Additional Information
Who can access the data: Any researchers whose proposed use of the data has been approved
Types of analyses: For any researchers whose proposed use of the data has been approved
Mechanisms of data availability: With a signed data access agreement
